# Inhibition of Adult Hippocampal Neurogenesis Plays a Role in Sevoflurane-Induced Cognitive Impairment in Aged Mice Through Brain-Derived Neurotrophic Factor/Tyrosine Receptor Kinase B and Neurotrophin-3/Tropomyosin Receptor Kinase C Pathways

**DOI:** 10.3389/fnagi.2022.782932

**Published:** 2022-03-04

**Authors:** Lichi Xu, Yanjing Guo, Gongming Wang, Guoqing Sun, Wei Sun, Jingjing Li, Xinlei Li, Jiangnan Wu, Mengyuan Zhang

**Affiliations:** ^1^Department of Anesthesiology, Shandong Provincial Hospital, Cheeloo College of Medicine, Shandong University, Jinan, China; ^2^Department of Anesthesiology, Shandong Provincial Hospital Affiliated to Shandong First Medical University, Jinan, China

**Keywords:** aging, sevoflurane, cognitive impairment, brain-derived neurotrophic factor, neurotrophin-3, adult hippocampal neurogenesis (AHN)

## Abstract

Sevoflurane anesthesia induces cognitive impairment, which may lead to perioperative neurocognitive disorders (PND). However, the factors and molecular mechanism underlying this impairment remains unclear. Adult hippocampal neurogenesis (AHN) in the subgranular zone of the hippocampus has been implicated in cognitive processes. Nonetheless, the direct role of AHN in sevoflurane-induced cognitive impairment has never been demonstrated. In this study, we explored the age and the concentration factors and the role of AHN inhibition in sevoflurane-induced cognitive impairment in sevoflurane inhalation model mice. We found that 3% sevoflurane exposure induced significant cognitive impairment and inhibition of AHN in aged mice but not adult mice. Expression of BDNF/TrkB and NT-3/TrkC was also decreased by 3% sevoflurane exposure in aged mice. Hippocampal brain-derived neurotrophic factor (BDNF) or Neurotrophin-3 (NT-3) microinjection could partially improve the sevoflurane-induced cognitive impairment and AHN inhibition, respectively. These results demonstrate that the cognitive impairment caused by sevoflurane inhalation is related to patient age and sevoflurane concentration. In conclusion, the molecular mechanism of cognitive impairment in the elderly is related to the inhibition of AHN through the BDNF/TrkB and NT-3/TrkC pathways. Thus, sevoflurane inhalation anesthesia may be safe for adult patients, but caution should be exercised when administering it to the elderly.

## Introduction

Nowadays, more and more elderly patients need surgical treatments. Sevoflurane is a commonly anesthetics for elderly patients which has many advantages such as easy-control, analgesia and muscle relaxation (Zhang Y. et al., 2018). However, sevoflurane tends to induce perioperative neurocognitive disorders (PND) which refer to the slight damage caused to higher cerebral cortical functions such as memory, concentration, and information processing capabilities that occur during the perioperative period (Bedford, 1955; Zhang Y. et al., 2018). PND prolongs the length of hospital stay and increases the cost of inpatient and out-of-hospital care. It affects the prognosis of patients and increases the postoperative mortality rate (Monk et al., 2008).

By now, reports on the effect of sevoflurane on cognition are conflicting (Monk et al., 2008; Herling et al., 2017; Tian et al., 2018; Yang and Yuan, 2018; Zhang H. et al., 2018; Zhang Y. et al., 2018). The different studies showed that many factors could influence the research conclusions above, such as different inhalation concentrations, times of exposure, periods of exposure, and brain backgrounds (Bedford, 1955; Monk et al., 2008; Herling et al., 2017; Tian et al., 2018; Yang and Yuan, 2018; Zhang H. et al., 2018; Zhang Y. et al., 2018). Clinical observation has showed that sevoflurane inhalation anesthesia could increase the incidence of PND in elderly patients (Zhang Y. et al., 2018). The basic studies have demonstrated that sevoflurane-induced cognitive impairment in young rats was dependent on the rats’ age and inhalation of 1.5% sevoflurane had no effect on cognition in adult rats but inhalation of both 2 and 3% sevoflurane produced significant cognitive impairment in aged rats (Shen et al., 2013; Tian et al., 2018; Yang and Yuan, 2018; Li et al., 2019).

Adult hippocampal neurogenesis (AHN) plays a key role in the recovery of learning and memory abilities after injury which occurs in the subgranular zone (SGZ) of the hippocampal dentate gyrus (DG) (Goncalves et al., 2016; Sailor et al., 2017). SGZ contains neural stem cells (NSCs) which differentiate into neural progenitor cells (NPCs) (Goncalves et al., 2016; Sailor et al., 2017). NPCs expressing doublecortin (DCX) differentiate into mature neurons, which are integrated into the granular layer (GL) of the DG. Therefore, DCX has been used as a marker for the analysis of AHN (Klein et al., 2020; Zhang et al., 2020). AHN is associated with the function of learning and memory of hippocampus (Wang W. X. et al., 2018); thus, inhibition of AHN could lead to cognitive impairment (Wang W. X. et al., 2018).

Brain-derived neurotrophic factor (BDNF) plays an important role in regulating AHN, which mainly affects the proliferation and differentiation of neurons in the hippocampus (Leal et al., 2017). BDNF specifically binds to tyrosine receptor kinase B (TrkB) in neural cells (Trzaska et al., 2009). Neurotrophin-3 (NT-3) exerts trophic effects on NPCs and premature neurons, which can promote neuronal differentiation, neurite outgrowth, synapse formation, and plasticity during AHN (Huang and Reichardt, 2003). NT-3 binds to tropomyosin receptor kinase C (TrkC), a neurotrophic factor receptor tyrosine kinase, and activates its tyrosine kinase function to drive the intracellular signal cascade to undergo the functions mentioned above (Shimazu et al., 2006).

Therefore, we have hypothesized that the effect of sevoflurane on cognitive function may be mainly influenced by sevoflurane concentration and patient age. In the present study, we have tested this hypothesis by identifying the different effects of sevoflurane concentration (1.5 vs. 3.0%) on cognitive impairment in different age groups (8 vs. 18-month-old) of mice. We assessed the effects of different sevoflurane concentrations on the numbers of DCX cells and 5-Bromodeoxyuridine (BrdU), proliferating cell marker, cells in the hippocampi of mice with different ages to explore the potential mechanism (Arvidsson et al., 2002; Thored et al., 2009). In view of the importance of the two neurotrophic factors above play in AHN, we assessed the effects of different sevoflurane concentrations on the levels of BDNF/TrkB and NT-3/TrkC in the hippocampi of mice with different ages. We also set an experiment of hippocampal BDNF/NT-3 microinjection to explore the potential mechanism. Through a series of *in vivo* studies, we demonstrated that sevoflurane-induced cognitive impairment in aged mice depended on the inhibition of AHN in the SGZ through the BDNF/TrkB and NT-3/TrkC pathways.

## Materials and Methods

### Ethical Approval

The use of mice in this study was approved by the Institutional Animal Care and Use Committee at Shandong Provincial Hospital (Jinan, China) (Ethical approval number: NSFC: No. 2019-167). All experiments were in accordance with the National Institutes of Health Guide for the Care and Use of Laboratory Animals and ARRIVE guidelines. Only male mice were used to exclude the effect of estrogen on the cognitive test data and biochemical data. The mice were housed in a 12-h:12-h light: dark cycle (light from 08:00 to 20:00) and the room temperature (RT) was maintained at 23 ± 1°C.

### Mice Anesthesia

Eight-month-old (adult) or eighteen-month-old (aged) C57BL/6 mice were randomly divided into control (CON group), 1.5% sevoflurane-inhaled (1.5% Sevo group), and 3% sevoflurane-inhaled (3.0% Sevo group). We placed mice in 1.5% Sevo group and 3.0% Sevo group in a plastic container and made them exposed to 1.5 or 3% Sevoflurane continuously for 3 h daily over 3 consecutive days (Shen et al., 2013). Sixty percent oxygen were used as a carrier, with a total gas flow of 2 L min^–1^. During exposure, we used a hot water bag on the bottom of the container with temperature maintained between 30 and 35°C to prevent low-body-temperature. Spontaneous respiratory frequency and skin color of ear and tail of mice were observed by an investigator every 10 min to judge apnea or hypoxia. Specifically, 8- and 18-month-old mice had a quick arterial blood sampling at the end of anesthesia to measure blood gas. A single sample was analyzed immediately after blood collection by using a blood gas analyzer. We analyzed pH, arterial carbon dioxide tension (PaCO_2_), arterial oxygen tension (PaO_2_), and blood glucose levels of arterial blood samples (Tables 1, 2).

**TABLE 1:** Arterial blood analysis of aged mice.

	CON	1.5% Sevo	3.0% Sevo	*p*-value
pH	7.40 ± 0.02	7.42 ± 0.02	7.42 ± 0.02	0.469
PaCO_2_(mmHg)	26.3 ± 1.18	25.4 ± 1.31	23.2 ± 1.02	0.190
PaO_2_(mmHg)	105.3 ± 4.73	103.2 ± 4.10	101.5 ± 5.24	0.853
Glucose (mmol⋅L^–1^)	5.2 ± 0.23	5.8 ± 0.26	5.7 ± 0.30	0.214

*Mean ± S.E.M, n = 7/group.*

**TABLE 2:** Arterial blood analysis of adult mice.

	CON	1.5% Sevo	3.0% Sevo	*p*-value
pH	7.39 ± 0.01	7.38 ± 0.01	7.42 ± 0.01	0.240
PaCO_2_(mmHg)	25.2 ± 0.55	23.3 ± 0.59	26.2 ± 0.98	0.272
PaO_2_(mmHg)	100.3 ± 4.66	101.2 ± 5.23	104.5 ± 4.92	0.825
Glucose (mmol⋅L^–1^)	5.8 ± 0.27	5.6 ± 0.28	5.2 ± 0.26	0.307

*Mean ± S.E.M, n = 7/group.*

### Behavioral Studies

#### Morris Water Maze Test

**FIGURE 1:**
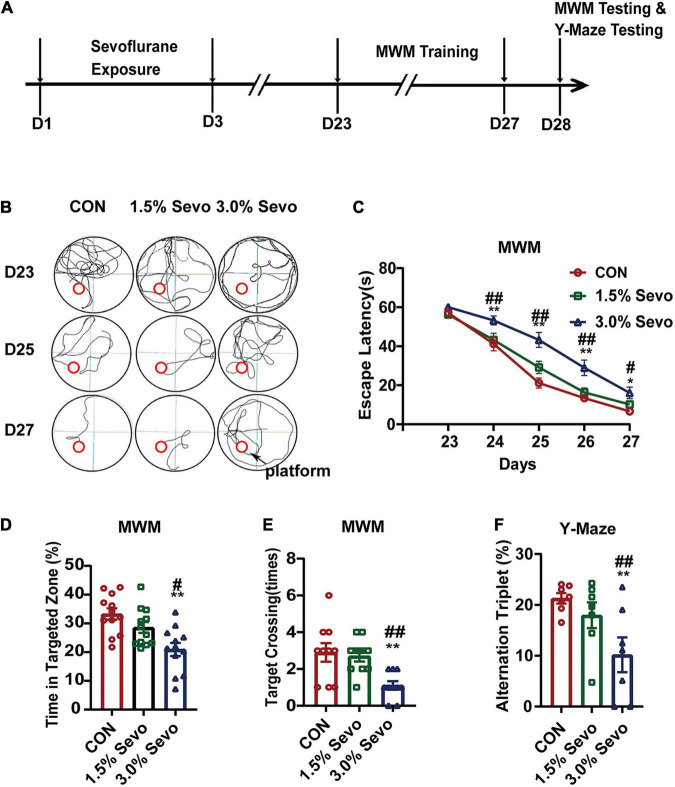
Exposure to 3% sevoflurane induces cognitive impairment in aged mice. **(A)** Timeline of the MWM and Y-Maze Test experimental procedure. **(B)** Representative tracks and **(C)** Escape latency of the MWM during training days. The platform is located in circle region. **(D)** The percentage of time in targeted zone in testing day. **(E)** Times of target crossing. **(F)** Alternation triplet of Y-maze. Values are the mean ± S.E.M. *p < 0.05, **p < 0.01, compared with the CON group. ^#^*p* < 0.05, ^##^*p* < 0.01 compared with the 1.5% Sevo group (*n* = 7/group). D, days of experimental procedure; MWM, Morris Water Maze Test; CON, control group; 1.5% Sevo, 1.5% sevoflurane group; 3.0% Sevo, 3.0% sevoflurane group.

Timeline of the MWM experimental procedure is placed in Figure 1A. In an opaque pool with a height of 60 cm and a diameter of 150 cm, there are 4 quadrants centered on the origin, northeast (NE), northwest (NW), southeast (SE), and southwest (SW). The pool was covered with a black curtain and was located in an isolated room. Each quadrant boundary (N, E, W, S) has a specific mark. Put a transparent platform (10 cm in diameter) in the center of SW quadrant. The water level is 1 cm higher than the platform. Water was kept at 20°C and opacified with bleaching powder. A video camera connected to a computer running tracking software was suspended above the pool and captured mice’s movements for analysis. Each mouse was put into the water from the boundary of 4 quadrants, and was counted for 60 s. Finding the platform (which can stay on the platform for more than 2 s) was recorded as the end of the experiment. If the platform cannot be found after 60 s, the experimenter would guide the mouse to the platform and stay on it for 15 s. Each mouse was tested in Morris water maze (MWM) four times (once per quadrant) per day for 5 days. Average escape latency time was measured to evaluate learning ability. The test is performed on the 6th day. At the end of the reference training, the platform was removed from the pool and each mouse was placed in the opposite quadrant (N and E). Each mouse was allowed to swim for 60 s. We calculated time spent in the zone that previously contained the platform and frequency of crossing the former location of the platform. Specifically, after every trial, each mouse was placed in a warm holding cage for at least 1–2 min to dry before being returned to its regular cage (Vorhees and Williams, 2006).

#### Y-Maze Test

Timeline of the Y-maze experimental procedure is placed in Figure 1A. Working memory and exploratory activity were measured using a Y-maze apparatus which consists three arms and a central area with arm length: 40 cm, arm bottom width: 3 cm, arm upper width: 13 cm, height of wall: 15 cm. Each mouse was placed in the central area to explore freely for 3 min. One alternation is defined as the mouse enters three different arms three times consecutively. The number of total entries into the arms and alterations were recorded. Working memory was calculated as number of correct alterations/number of total new arm entries, as described in a previous paper (Kraeuter et al., 2019; Yoshizaki et al., 2020).

### Bromodeoxyuridine Injections and Immunofluorescence

For BrdU injections, we followed the methods as previously mentioned. BrdU is a marker for proliferating cells that incorporates DNA in S-phase of mitosis (Arvidsson et al., 2002; Thored et al., 2009). BrdU (Sigma, America) was dissolved in normal saline (10 mg mL^–1^). On the first day of sevoflurane exposure, the mice started to be injected with BrdU intraperitoneally at a dosage of 100 mg/kg. BrdU injection was twice a day and lasted for 7 days (Zhang et al., 2020). One day after the last time of BrdU injection, those mice were anesthetized by intraperitoneal injection of pentobarbital to obtain brain tissue for immunofluorescence. Then we open each mouse’s thoracic cavity to expose heart. Right atrial appendage is cut with ophthalmic scissors, and physiological saline is infused through left ventricle. Later, left ventricle of each mouse was perfused with 4% paraformaldehyde. After transcardial perfusion, cranial cavity was opened to take out the whole brain which was put in paraformaldehyde for fixation overnight, cryoprotected in 20% sucrose, and then cut coronally in 9 μm thick sections on powdered dry ice. Sections were incubated in 1 M HCl at 65°C for 10 min and at room temperature for 20 min. Then, sections were blocked in 3% BSA and 0.1% Triton X-100 for 30 min at room temperature and incubated with primary antibody (mouse anti-BrdU, 1:200, rabbit anti-DCX, 1:200) in 1% BSA at 4°C over night. The sections were incubated with secondary antibody (Abcam goat anti-rabbit IgG, 1:1,000, Abcam goat anti-mouse IgG, 1:1,000) at room temperature for 1 h. We detected fluorescence with a fully automatic fluorescence microscope (OLYMPUS BX53M). All assessments were performed by an observer blind to the treatment conditions. The number of immunoreactive cells was counted in DG at 40 × magnification using an epifluorescence microscope in coronal sections. Representative immunofluorescence image of BrdU+DCX+ cell in hippocampal DG of aged mice exposed to 3% sevoflurane at 40 × magnification was shown in Supplementary Figure 1 as an example. All cells were counted in DG, both ipsi- and contralaterally. Six hippocampal slices per mouse were used to estimate the cells in the hippocampus. The image (1,600*1,200 pixels) of DCX+ cells, BrdU+ cells and BrdU+DCX+ cells was obtained at 20 × magnification (Arvidsson et al., 2002; Thored et al., 2009).

### Western Blot Analysis

Mice were anesthetized by intraperitoneal injection of pentobarbital for hippocampal tissue collection at the same day as immunofluorescence brain tissue collection. Then we open each mouse’s thoracic cavity to expose heart. Right atrial appendage is cut with ophthalmic scissors, and physiological saline is infused through left ventricle. Then cranial cavity was opened to take out the whole brain. We lift the cortex to expose the hippocampus under a stereo microscope. We separate hippocampal tissue and stored the tissue at −80°C for western blot. Protein was extracted by using RIPA lysis buffer. A protein analysis kit (BCA) was used to measure the protein content of each hippocampal tissue. We performed electrophoresis on a polyacrylamide SDS gel loaded with an average amount of 30 μg protein in each lane, and transfer the protein to PVDF membrane. The membrane was blocked with 5% skim milk in Tris buffered saline (TBST) for 1 h, and incubated with anti-BDNF (1:2,000), anti-TrkB (1:2,000), anti- NT-3 (1:2,000), anti-TrkC (1:2,000) primary antibodies at 4°C overnight. After rinsing, we probe the membrane with the corresponding secondary antibodies for 2 h at room temperature. An enhanced chemiluminescence detection system is used to detect the immune response bands. GAPDH and actin antibody is used to normalize the loading and transfer of samples. The intensity of the band was quantified by using the ImageJ optical density method.

### Hippocampal Microinjections

Twenty four hours after sevoflurane exposure, the mice were anesthetized using pentobarbital and we found the location of the hippocampal dentate gyrus on the surface of the skull by brain stereotaxic device. The location of the hippocampal dentate gyrus is according to the stereotaxic coordinates of mouse brains: AP–2.3 mm; ML ± 1.8 mm; DV-2 mm (Xiong et al., 2017). Tightly fitted screws were used to drill two skull screw holes. Micro syringes were used to deliver drugs. Mice received bilateral microinjection of BDNF (0.1 μg/side), NT-3 (0.025 μg/side) or saline (0.9%) into the dentate gyrus of hippocampus. A total volume of 1.0 μl was infused into each side over 15 min, and the injection syringe was left in place for an additional 5 min to allow for diffusion (Shirayama et al., 2002).

### Statistical Analysis

The results were expressed as mean ± S.E.M. The statistical tests were conducted using the computerized statistical package SPSS 25.0 (SPSS Inc., Chicago, IL, United States) and GraphPad Prism Software version 8.0 (GraphPad Software, Inc., San Diego, CA, United States). Interaction between time and group factors in a two-way ANOVA with repeated measurements was used to analyze the difference of learning curves (based on escape latency) of different groups in the MWM. Age and sevoflurane are two factors that affect cognitive function and AHN. We used two-way ANOVA to analyze the difference of other aspects of behavioral tests and the numbers of DCX+ cells, BrdU+ cells and BrdU+DCX+ cells among groups in aged and adult mice. One-way ANOVA was used to evaluate differences in the quantities of corresponding proteins in hippocampal tissue in aged or adult mice. For BDNF&NT-3 microinjection, we use one-way ANOVA to evaluate differences in the numbers of DCX+ cells, BrdU+ cells and BrdU+DCX+ cells among groups in aged mice. We used LSD-*t*-test for *post-hoc* comparison. The LSD-*t*-test is suitable for comparison between one or several pairs of sample means with special significance in the profession. Each experiment was performed at least three times. A value of *p* < 0.05 was considered statistically significant.

## Results

### Exposure to 3% Sevoflurane Induced Cognitive Impairment in Aged Mice but Not Adult Mice

To explore the effects of different doses of sevoflurane on cognitive function in mice of different ages, we established an animal model in which aged or adult mice were exposed to 1.5 or 3.0% sevoflurane for 3 h daily for 3 days. This animal model covered the low versus high concentration of anesthesia and allowed us to observe sevoflurane-induced neurotoxicity in brains of different ages.

The MWM Test, which is a method to assess spatial or place learning and memory, has been proven to be a robust and reliable test strongly correlated with hippocampal synaptic plasticity (Vorhees and Williams, 2006). Cognitive function was tested using MWM in aged mice (18-month-old) exposed to 1.5 or 3% sevoflurane for 3 h daily for 3 days. The escape latency is an index to test the learning ability, which refers to the time that each mouse spent to reach the platform during training. A statistical analysis between adult and aged mice was done to evaluate whether age impacts on sevoflurane-induced cognitive impairment. The results showed that both age and sevoflurane concentration are factors which could affects cognitive function (Table 3). The LSD-*t*-test multiple comparison showed that the escape latency increased significantly in aged mice exposed to 3% sevoflurane on experimental days 24–27 compared with that in mice of the 1.5% Sevo (ΔSD = 9.26, *n* = 7/group, *p* = 0.001) or CON group (ΔSD = 12.39, *n* = 7/group, *p* = 0.001) (Figures 1B,C). The LSD-*t*-test multiple comparison showed that the time spent in targeted zone was significantly decreased in aged mice in the 3% Sevo group (20.9 ± 2.27) compared with that in aged mice in 1.5% Sevo (28.6 ± 1.90, ΔSD = −7.66, *n* = 7/group, *p* = 0.013) or CON (33.2 ± 2.00, ΔSD = −12.32, *n* = 7/group, *p* = 0.001) group (Figure 1D). The times of target-crossing was significantly decreased in aged mice in the 3% Sevo group (1.1 ± 0.23) compared with that in aged mice in 1.5% Sevo (2.7 ± 0.30, ΔSD = −14.76, *n* = 7/group, *p* = 0.001) or CON (2.9 ± 0.50, ΔSD = −18.09, *n* = 7/group, *p* = 0.001) group (Figure 1E). There is no significant difference in swimming speed in MWM test in aged (*F* = 1.58, *n* = 7/group, *p* = 0.219) and adult (*F* = 0.170, *n* = 7/group, *p* = 0.844) mice (Supplementary Table 1). Additionally, spatial reference memory, which is hippocampus-dependent, can be tested by placing the test mice into a Y-maze with one arm closed off during training (Kraeuter et al., 2019; Yoshizaki et al., 2020). The ratio of alternation triplet of Y-maze decreased in the 3% Sevo group (10.19 ± 3.44) compared with that in aged mice in 1.5% Sevo (24.95 ± 2.52, ΔSD = −14.76, *n* = 7/group, *p* = 0.001) or CON (28.29 ± 1.02, ΔSD = −18.09, *n* = 7/group, *p* = 0.001) group (Figure 1F). There was no significant difference in the MWM and Y-maze tests between mice in the CON and 1.5% Sevo groups.

**TABLE 3:** Two-way ANOVA analysis of cognitive function in adult and aged mice.

	Age	Sevo
Escape latency*	*F* = 278.1, *p* = 0.001	*F* = 13.88, *p* = 0.001
Time spent in targeted zone	*F* = 4.546, *p* = 0.036	*F* = 4.269, *p* = 0.018
Target-crossing	*F* = 9.131, *p* = 0.004	*F* = 3.532, *p* = 0.035
Alternation triplet	*F* = 9.418, *p* = 0.004	*F* = 1.614, *p* = 0.212

*n = 7/group.*

*Two-way ANOVA with repeated measurements was used to analyze the difference of learning curves (based on escape latency) of different groups in the MWM. Two-way ANOVA was used to analyze other aspects of behavior test.

**FIGURE 2:**
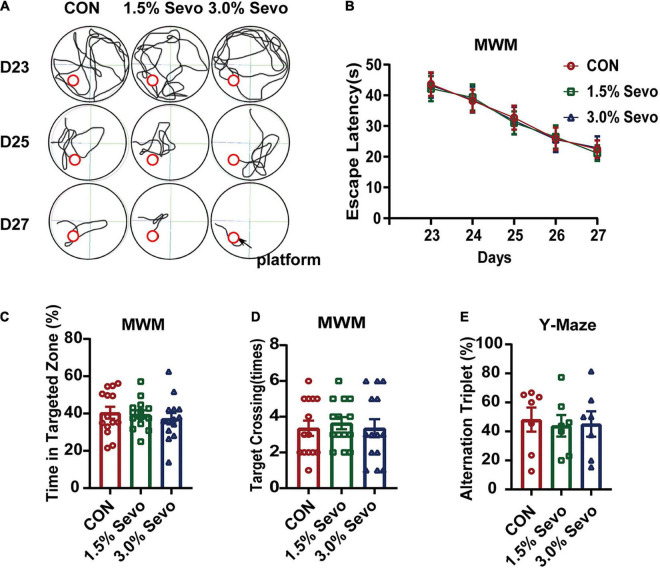
Exposure to 1.5 or 3% sevoflurane does not induce cognitive impairment in adult mice. **(A)** Representative tracks and **(B)** escape latency of the MWM during training days. The platform is located in circle region. **(C)** The percentage of time in targeted zone in testing day. **(D)** Times of target crossing. **(E)** Alternation triplet of Y-maze. Values are the mean ± S.E.M (*n* = 7/group). D, days of experimental procedure; MWM, Morris Water Maze Test; CON, control group; 1.5% Sevo, 1.5% sevoflurane group; 3.0% Sevo, 3.0% sevoflurane group.

To further dissect the role of age in sevoflurane-induced cognitive impairment, adult mice (8-month-old) were exposed to 1.5 or 3% sevoflurane for 3 h daily for 3 days and cognitive function was tested using MWM and Y-maze. There was no significant difference in escape latency (Figures 2A,B), time spent in targeted zone (Figure 2C), times of target-crossing (Figure 2D) of MWM, or ratios of alternation triplet (Figure 2E) of the Y-maze.

### Exposure to 3% Sevoflurane Induced Inhibition of Adult Hippocampal Neurogenesis in Aged Mice

**FIGURE 3:**
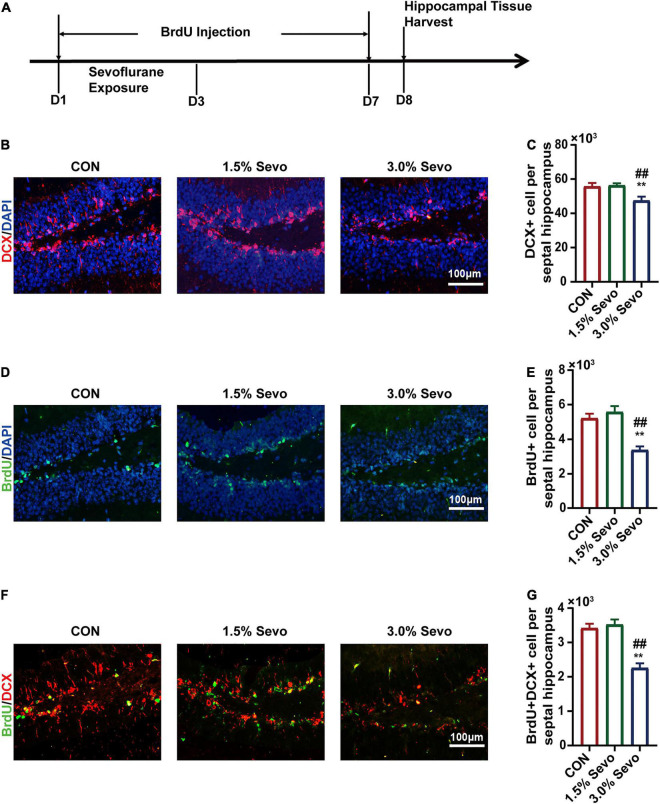
The inhibition of DCX+ cells, BrdU+ cells, and BrdU+DCX+ cells is induced by 3% sevoflurane in the hippocampi of aged mice. **(A)** Timeline of the immunofluorescence experimental procedure. **(B)** Representative immunofluorescence images of DCX+ cells in aged mice. **(C)** Quantitative analysis of DCX+ cells in aged mice. **(D)** Representative immunofluorescence images of BrdU+ cells in aged mice. **(E)** Quantitative analysis of BrdU+ cells in aged mice. **(F)** Representative immunofluorescence images of BrdU+DCX+ cells in aged mice. **(G)** Quantitative analysis of BrdU+DCX+ cells in aged mice. Values are the mean ± S.E.M. **p < 0.01 compared with the CON group. ^##^*p* < 0.01 compared with the 1.5% Sevo group (*n* = 8/group). D, days of experimental procedure; BrdU, 5-Bromodeoxyuridine; DCX, doublecortin; CON, control group; 1.5% Sevo, 1.5% sevoflurane group; 3.0% Sevo, 3.0% sevoflurane group.

**FIGURE 4:**
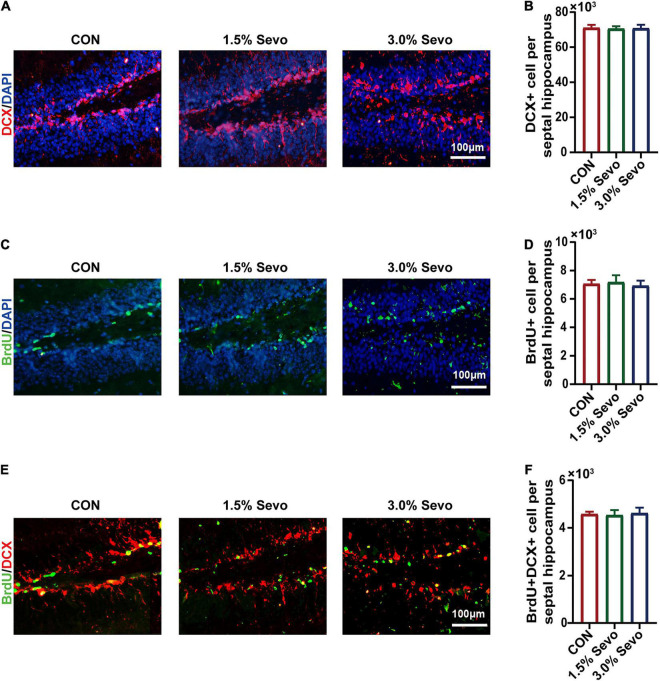
Sevoflurane exposure does not induce the inhibition of DCX+, BrdU+ and BrdU+DCX+ cells in the hippocampi of adult mice. **(A)** Representative immunofluorescence images of DCX+ cells in adult mice. **(B)** Quantitative analysis of DCX+ cells in adult mice. **(C)** Representative immunofluorescence images of BrdU+ cells in adult mice. **(D)** Quantitative analysis of BrdU+ cells in adult mice. **(E)** Representative immunofluorescence images of BrdU+DCX+ cells in adult mice. **(F)** Quantitative analysis of BrdU+DCX+ cells in adult mice. Values are the mean ± S.E.M (*n* = 8/group). BrdU, 5-Bromodeoxyuridine; DCX, doublecortin; CON, control group; 1.5% Sevo, 1.5% sevoflurane group; 3.0% Sevo, 3.0% sevoflurane group.

To evaluate whether the cognitive impairment caused by 3% sevoflurane was related to AHN in aged mice, immunofluorescence of BrdU and DCX was performed, which are markers of newborn neural cells and NPCs, respectively (Figure 3A). Representative images of the effects of sevoflurane inhalation in newborn neural cells (BrdU+) and NPCs (DCX+) are shown in Figures 3B,D, 4A,C. A statistical analysis between adult and aged mice was done to evaluate whether age impacts on sevoflurane-induced inhibition of AHN by using two-way ANOVA. The results showed that both age and sevoflurane concentration are factors which could affects AHN (Table 4). The LSD-*t*-test multiple comparison showed that compared with the CON (55,743 ± 1,905, ΔSD = −8,252.3, *n* = 8/group, *p* = 0.006) and 1.5% Sevo (56,334 ± 1,141, ΔSD = −8844.0, *n* = 8/group, *p* = 0.003) groups, the 3% Sevo group (47,490 ± 2,119) showed significantly decreased number of DCX+ cells in aged mice (Figure 3C). The 3% Sevo group (3,886 ± 188) showed significantly decreased number of BrdU+ cells compared with the CON (5,225 ± 240, ΔSD = −4,598.6, *n* = 8/group, *p* = 0.001) and 1.5% Sevo (5,584 ± 314, ΔSD = −5,494.2, *n* = 8/group, *p* = 0.001) groups in aged mice (Figure 3E). To evaluate the effects of sevoflurane on neuronal commitment of proliferating NSCs, BrdU+DCX+ was used to detect newborn NPCs. Representative images of sevoflurane effects in BrdU+DCX+ cells are shown in Figures 3F, 4E. According to the results, 3% sevoflurane (2,257 ± 126) exposure significantly decreased the number of BrdU+DCX+ cells compared with the CON (3,417 ± 120, ΔSD = −2,899.1, *n* = 8/group, *p* = 0.001) and 1.5% Sevo (3,522 ± 138, ΔSD = −3,162.8, *n* = 8/group, *p* = 0.001) groups in aged mice (Figure 3G), suggesting that 3% sevoflurane exposure inhibited the proliferation of newborn NPCs. There was no significant difference in cell numbers between the mice in the control and 1.5% sevoflurane groups.

**TABLE 4:** Two-way ANOVA analysis of AHN in adult and aged mice.

	Age	Sevo
DCX+ cells	*F* = 129.8, *p* = 0.001	*F* = 3.39, *p* = 0.042
BrdU+ cells	*F* = 63.95, *p* = 0.001	*F* = 6.62, *p* = 0.003
BrdU+DCX+ cells	*F* = 90.06, *p* = 0.001	*F* = 5.79, *p* = 0.006

*n = 8/group.*

To determine whether cognitive impairment and the inhibition of AHN occur simultaneously, AHN was also evaluated in adult mice. Results are given in the Figure 4. There were no significant differences in the number of BrdU+, DCX+, or BrdU+DCX+ cells among the three groups of adult mice (Figures 4B,D,F), indicating that sevoflurane exposure did not lead to AHN inhibition in adult mice.

### Sevoflurane (3%) Exposure in Aged Mice Induced Downregulation of Hippocampal Neurotrophic Factors Brain-Derived Neurotrophic Factor/Tyrosine Receptor Kinase B and Neurotrophin-3/Tropomyosin Receptor Kinase C

**FIGURE 5:**
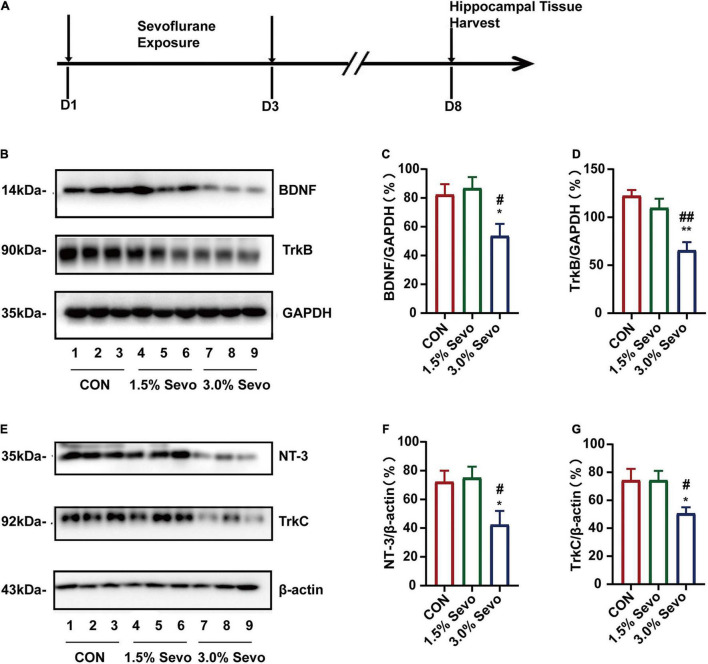
Sevoflurane exposure induces the reduction of BDNF/TrkB and NT-3/TrkC in the hippocampi of aged mice. **(A)** Timeline of the western blot experimental procedure. **(B)** Representative WB images of BDNF and TrkB expression in aged mice. Quantitative analysis of BDNF **(C)** and TrkB **(D)**. GAPDH was used as an internal standard. **(E)** Representative WB images of NT-3 and TrkC expression in aged mice. Quantitative analysis of NT-3 **(F)** and TrkC **(G)**. β-actin was used as an internal standard. Values are the mean ± S.E.M. *p < 0.05, **p < 0.01 compared with the CON group. ^#^*p* < 0.05, ^##^*p* < 0.01 compared with the 1.5% Sevo group (*n* = 9/group). D, days of experimental procedure; BDNF, brain-derived neurotrophic factor; TrkB, tyrosine receptor kinase B; NT-3, Neurotrophin-3; TrkC, tyrosine receptor kinase C; CON, control group; 1.5% Sevo, 1.5% sevoflurane group; 3.0% Sevo, 3.0% sevoflurane group.

Neurotrophic factors and their corresponding receptors, particularly BDNF/TrkB and NT-3/TrkC, are associated with cognitive impairment and AHN (Rossi et al., 2006; Shimazu et al., 2006). In order to explore the potential mechanisms of sevoflurane-induced cognitive impairment and AHN inhibition, we evaluated the effects of sevoflurane exposure on the expression levels of BDNF/TrkB and NT-3/TrkC in the hippocampal tissues of aged mice. The hippocampal tissues were harvested and subjected to western blot analysis to determine the levels of BDNF/TrkB and NT-3/TrkC (Figure 5A). Representative western blots are shown in Figures 5B,E. The results showed that 3% sevoflurane exposure significantly decreased the levels of BDNF (*F* = 5.46, *n* = 9/group, *p* = 0.011)/TrkB (*F* = 13.5, *n* = 9/group, *p* = 0.001) and NT-3 (*F* = 4.74, *n* = 9/group, *p* = 0.018)/TrkC (*F* = 5.05, *n* = 9/group, *p* = 0.015) in aged hippocampal tissues (Figures 5C,D,F,G). There were no significant differences in BDNF/TrkB or NT-3/TrkC levels between the aged mice of the 1.5% Sevo and CON groups.

**FIGURE 6:**
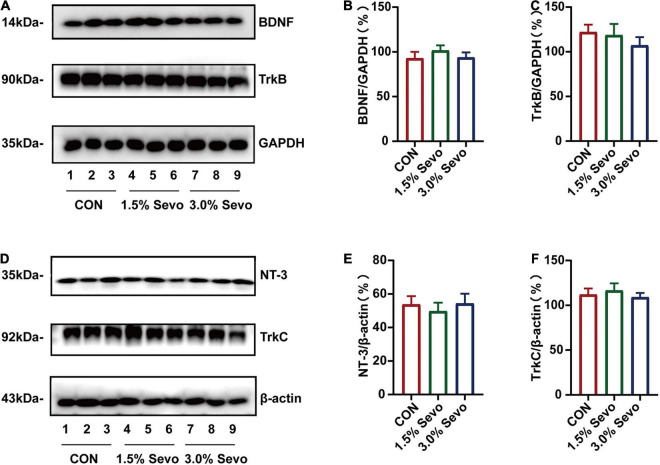
Sevoflurane exposure does not induce the reduction of BDNF/TrkB and NT-3/TrkC in the hippocampi of adult mice. **(A)** Representative WB images of BDNF and TrkB expression in adult mice. Quantitative analysis of BDNF **(B)** and TrkB **(C)**. GAPDH was used as an internal standard. **(D)** Representative WB images of NT-3 and TrkC expression in adult mice. Quantitative analysis of NT-3 **(E)** and TrkC **(F)**. β-actin was used as an internal standard. Values are the mean ± S.E.M (*n* = 9/group). BDNF, brain-derived neurotrophic factor; TrkB, tyrosine receptor kinase B; NT-3, Neurotrophin-3; TrkC, tyrosine receptor kinase C; CON, control group; 1.5% Sevo, 1.5% sevoflurane group; 3.0% Sevo, 3.0% sevoflurane group.

To evaluate the relationship between sevoflurane-induced neurotrophin inhibition and age, adult mice were subjected to sevoflurane exposure. Sevoflurane exposure did not decrease the levels of BDNF (*F* = 0.587, *n* = 9/group, *p* = 0.564)/TrkB (*F* = 0.586, *n* = 9/group, *p* = 0.564) (Figures 6B,C) or NT-3 (*F* = 0.225, *n* = 9/group, *p* = 0.800)/TrkC (*F* = 0.385, *n* = 9/group, *p* = 0.685) (Figures 6E,F) in the hippocampal tissues of adult mice. Taken together, these results suggest that in aged mice, 3% sevoflurane exposure decreases neurotrophic factor levels in the hippocampal tissues, possibly leading to AHN inhibition and cognitive impairment.

### The Cognitive Impairment and the Inhibition of Adult Hippocampal Neurogenesis Caused by Sevoflurane Are Related to Brain-Derived Neurotrophic Factor/Tyrosine Receptor Kinase B and Neurotrophin-3/Tropomyosin Receptor Kinase C Pathways

**FIGURE 7:**
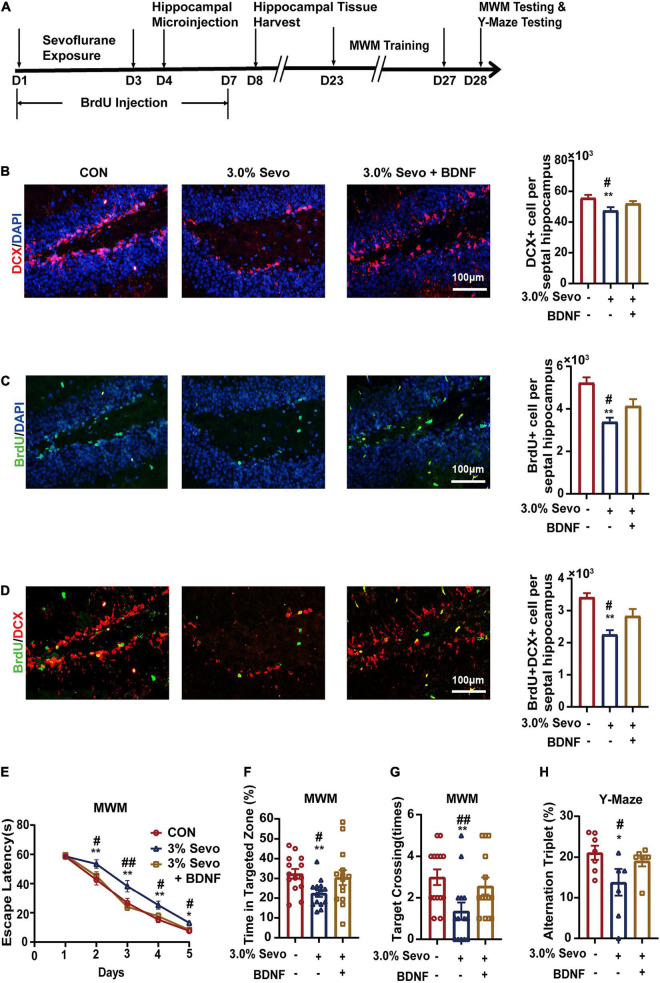
Cognitive impairment and inhibition of AHN induced by 3% sevoflurane in aged mice could be partially prevented by BDNF. **(A)** Timeline of hippocampal microinjection procedure. **(B)** Representative immunofluorescence images and quantitative analysis of DCX+ cells in aged mice. **(C)** Representative immunofluorescence images and quantitative analysis of BrdU+ cells in aged mice. **(D)** Representative immunofluorescence images and quantitative analysis of BrdU+DCX+ cells in aged mice. **(E)** Escape latency of the MWM during training days. **(F)** The percentage of time in targeted zone in testing day. **(G)** Times of target crossing. **(H)** Alternation triplet of Y-maze. Values are the mean ± S.E.M. *p < 0.05, **p < 0.01 compared with the CON group. ^#^*p* < 0.05, ^##^*p* < 0.01 compared with the BDNF group (*n* = 8/group for immunofluorescence and *n* = 7/group for behavioral test). D, days of experimental procedure; MWM, Morris Water Maze Test; BrdU, 5-Bromodeoxyuridine; DCX, doublecortin; BDNF, brain-derived neurotrophic factor; TrkB, tyrosine receptor kinase B; NT-3, Neurotrophin-3; TrkC, tyrosine receptor kinase C; CON, control group; 1.5% Sevo, 1.5% sevoflurane group; 3.0% Sevo, 3.0% sevoflurane group.

**FIGURE 8:**
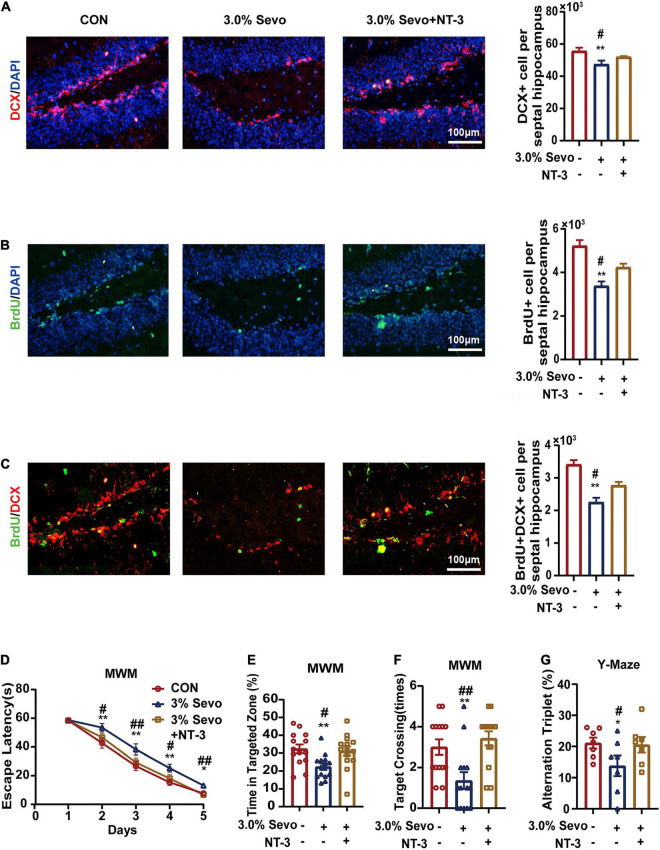
Cognitive impairment and inhibition of AHN induced by 3% sevoflurane in aged mice could be partially prevented by NT-3. **(A)** Representative immunofluorescence images and quantitative analysis of DCX+ cells in aged mice. **(B)** Representative immunofluorescence images and quantitative analysis of BrdU+ cells in aged mice. **(C)** Representative immunofluorescence images and quantitative analysis of BrdU+DCX+ cells in aged mice. **(D)** Escape latency of the MWM during training days. **(E)** The percentage of time in targeted zone in testing day. **(F)** Times of target crossing. **(G)** Alternation triplet of Y-maze. Values are the mean ± S.E.M. *p < 0.05, **p < 0.01 compared with the CON group. ^#^*p* < 0.05, ^##^*p* < 0.01 compared with the NT-3 group (*n* = 8/group for immunofluorescence and *n* = 7/group for behavioral test). MWM, Morris Water Maze Test; BrdU, 5-Bromodeoxyuridine; DCX, doublecortin; BDNF, brain-derived neurotrophic factor; TrkB, tyrosine receptor kinase B; NT-3, Neurotrophin-3; TrkC, tyrosine receptor kinase C; CON, control group; 1.5% Sevo, 1.5% sevoflurane group; 3.0% Sevo, 3.0% sevoflurane group.

BDNF and NT-3 have been reported to improve cell proliferation and differentiation in AHN (Huang and Reichardt, 2003; Shimazu et al., 2006; Frielingsdorf et al., 2007). We wondered whether BDNF/NT-3 could attenuate the cognitive impairment and AHN inhibition mediated by 3% sevoflurane in aged mice. The mice were given bilateral microinjections of BDNF or NT-3 into the hippocampus 24 h after sevoflurane exposure (Figures 7, 8). The representative images of newborn neural cells (BrdU+) (CON group 5,196 ± 236, 3.0% Sevo group 3,401 ± 195), NPCs (DCX+) (CON group 55,922 ± 1,891, 3.0% Sevo group 47,218 ± 2,006), and newborn NPCs (BrdU+DCX+) (CON group 3,453 ± 108, 3.0% Sevo group 2,301 ± 138) are shown in Figures 7B–D, 8A–C. Statistical analysis of AHN and cognitive function was shown in Supplementary Tables 2, 3. The results showed that hippocampal microinjection of BDNF and NT-3 significantly increased the number of DCX+ (BDNF group 52,106 ± 1,482, NT-3 group 52,225 ± 390), BrdU+ (BDNF group 4,132 ± 305, NT-3 group 4,242 ± 152), and BrdU+DCX+ (BDNF group 2,835 ± 203, NT-3 group 2,775 ± 101) cells, which were decreased in aged mice exposed to 3% sevoflurane (Figures 7B–D, 8A–C and Supplementary Table 2). The cognitive function tested by MWM and Y-Maze which is inhibited by 3% sevoflurane is also improved by hippocampal BDNF & NT-3 microinjection (Figures 7E–H, 8D–G and Supplementary Table 3). It is worth noting that the numbers of DCX+, BrdU+, and BrdU+DCX+ cells in the BDNF/NT-3 group were not as many as those in the CON group, which indicates that BDNF and NT-3 are both associated with AHN inhibition caused by sevoflurane (Figures 7B–D, 8A–C). These results suggest that the cognitive impairment and the inhibition of AHN caused by sevoflurane is related to the BDNF/TrkB and NT-3/TrkC pathways.

## Discussion

Sevoflurane is one of the most commonly used anesthetics in clinical practice. In this study, aged mice that were given 1.5% sevoflurane inhalation and adult mice exposed to sevoflurane did not suffer cognitive impairment. However, exposure of aged mice to 3% sevoflurane led to significant cognitive impairment, which may be due to the inhibition of AHN probably through the BDNF/TrkB and NT-3/TrkC pathways. These findings suggest that in clinical settings, sevoflurane inhalation anesthesia may be safe for adult patients, while caution should be used for the elderly.

Previous studies have proved that sevoflurane has adverse effects on developing brain such as fetal mice, newborn mice or young mice (Fang et al., 2012; Shen et al., 2013; Wang et al., 2019; Jia et al., 2020). It has been confirmed that sevoflurane exposure of developing brain tend to suffer cognitive impairment in adulthood such as learning and memory deficiency (Shen et al., 2013; Yu et al., 2020). Clinical study has shown that the proportion of elderly patients receiving sevoflurane anesthesia has increased significantly and sevoflurane anesthesia in elderly patients tend to cause cognitive dysfunction (Zhang Y. et al., 2018). However, the mechanism is unclear. Previous studies show that the effect of sevoflurane on cognitive function of elderly animals is controversial (Callaway et al., 2012; Liu et al., 2015; Hu et al., 2016; Yang and Yuan, 2018). Due to the complexity of clinical anesthesia and the difference between clinical anesthesia and basic research, we adopted the sevoflurane exposure method referring to Shen et al. (2013) and Luo et al. (2021) study in order to control the experimental factors.

Sevoflurane has side effects on respiration and circulation, which could lead to an acid-base imbalance or hypoxemia. Both acid-base imbalance and hypoxemia could induce brain injury which directly causes cognitive impairment (Vlisides and Xie, 2012; Zhang et al., 2019). Therefore, we conducted arterial blood gas analysis to confirm that sevoflurane exposure did not induce hypoxemia or acid-base imbalance. Results indicated that there were no significant differences in the blood gas parameters among groups in aged mice and adult mice, suggesting that sevoflurane exposure under our experimental conditions did not induce hypoxemia or significant acid-base imbalance (Tables 1, 2). Thus, the behavioral changes observed after sevoflurane exposure are likely not due to respiratory or cardiovascular complications.

MWM is a classical behavior test that is used to evaluate spatial reference memory (escape latency) and spatial learning memory (times spent in target zone and target crossing times), and has proven to be a robust and reliable test strongly correlated with hippocampal synaptic plasticity (Vorhees and Williams, 2006). The Y maze can detect the animal’s ability to distinguish spatial position and orientation, spatial working memory (short-term memory), and fragmented memory (Kraeuter et al., 2019; Yoshizaki et al., 2020). We used two behavioral tests to evaluate the different aspects of cognitive functions that may be affected by sevoflurane, and as we mentioned below, sevoflurane could cause cognitive impairment in many aspects. Through MWM and Y maze tests, we found that 3% sevoflurane exposure in aged mice caused cognitive impairment of spatial memory and spatial exploration. However, 1.5% sevoflurane exposure did not have any effect. The effects of sevoflurane exposure on cognition are age-dependent and concentration-dependent. According to our results and previous studies, sevoflurane tends to induce cognitive impairment in developing brain and aged brain (Fang et al., 2012; Zhang et al., 2013; Xu et al., 2018; Wang et al., 2019). At the same time, sevoflurane does not have a harmful effect on the cognitive function of the adult brain and it may have a beneficial effect under certain conditions (Liu et al., 2015; Xu et al., 2018). Developing brain and aged brain are fragile brains which are more susceptible to external harmful stimuli than adult brain (Y. Zhao et al., 2016; Gong et al., 2020; Yu et al., 2020). The mechanisms are related to neurogenesis, autophagy, Tau protein deposition, neuroinflammation, neuronal excitability, etc. (Fang et al., 2012; Zhang et al., 2013; Zhao et al., 2016; Xu et al., 2018; Wang et al., 2019; Gong et al., 2020; Yu et al., 2020). The effect of sevoflurane on cognition is also related to the dose. There is no effect or beneficial effect at low dose and cognitive impairment is induced at high dose (Fang et al., 2012; Zhang et al., 2013; Chen et al., 2018; Wang et al., 2019; Qin et al., 2020). Thus, only high sevoflurane concentration could induce cognitive impairment in aged mice. These findings indicate that sevoflurane exposure may have different cognitive effects on different brain ages, and only certain sevoflurane concentrations can induce cognitive impairment in mice of specific ages. These findings support the following clinical observation: Sevoflurane inhalation anesthesia at higher concentrations in aged patients undergoing surgery is likely to increase the risk of cognitive impairment during the perioperative period (Liu et al., 2013; Zhang Y. et al., 2018).

AHN is the growth of NSCs into NPCs, which finally turn into mature neurons throughout life. AHN plays an important role in various cognitive processes (Zhao et al., 2008; Goncalves et al., 2016). The SGZ of the hippocampal DG is one of the main germinal zones of adult rodents (Gage, 2000; Li and Pleasure, 2010). BrdU is used as a proliferating cell marker to detect AHN (Arvidsson et al., 2002; Thored et al., 2009). During AHN, NSCs produce NPCs that differentiate into neurons that functionally integrate into the GL in the SGZ of the hippocampal DG (Kempermann et al., 2015; Berg et al., 2018). DCX is the cell marker referring to NPCs (Klein et al., 2020; Zhang et al., 2020). Owing to their expression patterns, BrdU and DCX have become two widely used markers for the analysis of AHN (Brown et al., 2003; Couillard-Despres et al., 2005). The existence of AHN can be observed not only in adult/aged humans with normal cognitive function, but also in patients with mild cognitive impairment and Alzheimer’s disease (Moreno-Jiménez et al., 2019; Tobin et al., 2019). Kuhn et al. (1996) first quantified age-related decline in AHN in old rats through BrdU labeling and IHC analysis. It has been reported that sevoflurane has a dual effect on neurogenesis in young mice (Chen et al., 2015, 2018; Jia et al., 2020). However, there is lack of research to evaluate the effect of sevoflurane on AHN in adult or aged subjects. It has been proved that aging is a negative factor of AHN and aged brain lack of AHN is more likely to suffer cognitive impairment (Zhao et al., 2008). According to our experimental results, it can also be proved that AHN in aged mice is weaker than that in adult mice (Supplementary Table 2). Control groups in aged and adult mice were set up respectively, to analyze the effect of different doses of sevoflurane in different ages of mice. Our immunofluorescence results showed that sevoflurane has an inhibitory effect on the proliferation of NPCs of SGZ in the hippocampal DG. This phenomenon was not observed in adult mice, and aged mice exposed to 1.5% sevoflurane. These results indicate that sevoflurane has a dual effect on cognitive function and AHN depending on its dose and the age of exposed subjects, and cognitive impairment caused by sevoflurane may be related to the inhibition of AHN in the aged hippocampus.

Studies have reported that there is a correlation between AHN and cognition and the decline of AHN is one of the possible mechanisms of neurodegenerative disease (Li and Pleasure, 2010; Goncalves et al., 2016; Moreno-Jiménez et al., 2019; Tobin et al., 2019). However, the specific relationship between the decline of AHN and cognitive dysfunction (like AD) is still an open question (Li Puma et al., 2020). Jia et al. (2020) reported that the inhibition of neurogenesis was associated with cognitive impairment 4 weeks after sevoflurane exposure which is the same time point as we focus on. We focused on the cognitive decline diagnosed up to 30 days after surgery which is defined as “delayed neurocognitive recovery” and first observed that the cognitive impairment in aged mice 4 weeks after exposed to sevoflurane may be related to AHN inhibition (Evered et al., 2018).

Our results showed that sevoflurane exposure could induce AHN inhibition in aged mice, but not in adult mice. The mechanism may be related to neurotrophic factors. The expression of neurotrophic factors is related to AHN (Goncalves et al., 2016). Both BDNF and NT-3 belong to neurotrophic factors that play an important role in regulating hippocampal neurogenesis and cognitive function (Huang and Reichardt, 2003; Duman and Monteggia, 2006; Shimazu et al., 2006; Frielingsdorf et al., 2007; Zhao et al., 2008; Trzaska et al., 2009; Leal et al., 2017). In our study, 3% sevoflurane inhibited AHN and the expression of BDNF/NT-3 in the aged hippocampus simultaneously. Previous study has confirmed that the cognitive impairment induced by sevoflurane and the decrease of BDNF expression occur in parallel, and over-expression of BDNF could be prevented it (Xu and Qian, 2020). However, our study found that the way of BDNF regulating sevoflurane-induced cognitive impairment is AHN. It is well known that activities in the hippocampus can stimulate the transcription and translation of BDNF genes and BDNF regulates the development of neural cells and synaptic transmission (Zhao et al., 2008). Duman and Monteggia (2006) showed that changes in neurogenesis in the SGZ correlated with the expression of BDNF. Our findings provide more evidence for the interaction between AHN and BDNF/TrkB pathway. NT-3 exerts trophic effects on premature neurons, which include fate specification of neural cells, neurite outgrowth, synapse formation, and plasticity (Huang and Reichardt, 2003). There are studies showing that the spatial memory of NT-3 gene-mutant mice is significantly reduced and that NT-3 gene deficiency have a smaller number of newly differentiated neurons in the DG of the hippocampus (Shimazu et al., 2006; Frielingsdorf et al., 2007). Studies have showed that sevoflurane could cause downregulation of neurotrophic factors in developing brain (Tang et al., 2020; Zhao et al., 2021). As is mentioned above, like the developing brain, the aged brain is fragile brain which is more susceptible to external harmful factors compared with adult brain (Wang Z. et al., 2018; Xu et al., 2018; Yang and Yuan, 2018; Gong et al., 2020). Therefore, sevoflurane induces AHN inhibition and BDNF/NT-3 downregulation in aged mice while it has no effect on AHN and BDNF/NT-3 expression in adult mice. More importantly, we reported that the NT-3/TrkC pathway also played a role in the cognitive impairment and AHN inhibition induced by sevoflurane in aged mice for the first time. Our findings added new insight into the mechanism underpinning cognitive impairment in the elder subjected to sevoflurane anesthetic.

As explained above, AHN is a consistent process in the SGZ of the hippocampus, which consists of quiescence, proliferation/activation, fate specification, and mutation. DCX+ cells represent neural cells that undergo proliferation/activation, fate specification, and morphogenesis/differentiation during the process of AHN (Zhao et al., 2008; Goncalves et al., 2016). BDNF and NT-3 are two neurotrophic factors that mainly affect different stages of the AHN process. NT-3 mainly stimulates fate specification, while BDNF mainly stimulates morphogenesis/differentiation (Huang and Reichardt, 2003; Shimazu et al., 2006; Frielingsdorf et al., 2007; Zhao et al., 2008; Goncalves et al., 2016). Hence, microinjection of one of the two neurotrophic factors could not completely prevent the cognitive impairment and inhibition of AHN caused by sevoflurane, which also indicates that DCX+ cells may be target cells of both BDNF and NT-3 and cognitive impairment does not only depend on the number of DCX+ cells. In summary, the potential mechanism of sevoflurane-induced cognitive impairment is associated with the inhibition of AHN in SGZ which may involve the BDNF/TrkB and NT-3/TrkC pathways.

This study has several limitations. Firstly, we did not study the effects of anesthesia on other aspects of cognitive dysfunction. We focused on learning and memory functions and spatial exploration functions, which are the main aspects of cognitive dysfunction. Secondly, we only evaluated the effects of anesthesia in 18- and 8-month-old mice. It is unclear whether cognitive impairment and AHN inhibition caused by sevoflurane also occur in mice of other ages. Therefore, future research will include evaluating the effects of different concentrations of sevoflurane in mice of different ages (14, 16, and 20 months) to test the hypothesis that the elderly brain is more susceptible to sevoflurane neurotoxicity. Thirdly, there are some other factors affecting cognitive function such as neuronal viability and the survival of NPCs which are related to the pathogenesis of neurodegenerative diseases (Agostinho et al., 2010; Lopes and Agostinho, 2011; Zhang et al., 2020). Experiments will be set up to clear the specific influence of sevoflurane on neuronal viability and the survival of NPCs in aged mice. Finally, current data indicate that exposure to high concentrations of sevoflurane in aged mice may cause cognitive impairment and AHN inhibition, which may be related to the BDNF/TrkB and NT-3/TrkC pathways. AHN is a complicated process in the hippocampus and the relationship between the decline of AHN and cognitive dysfunction in AD or PND is still an open question (Fang et al., 2012; Li Puma et al., 2020). Thus, the specific stage and the accurate mechanism of AHN inhibited by sevoflurane remains to be determined.

In summary, we found that the sevoflurane-induced cognitive impairment is influenced by the concentration of sevoflurane and patient age. Taken together, these findings support the conclusion that inhibition of AHN at least partially lead to cognitive impairment caused by sevoflurane, which is probably attributed to the downregulation of BDNF/TrkB and NT-3/TrkC pathways. These findings should prompt further research to investigate anesthetic neurotoxicity in the fragile brains of elderly patients. Finally, it should provide safer anesthesia care and better postoperative results for elderly patients who may be vulnerable to brain injury.

## Data Availability Statement

The original contributions presented in the study are included in the article/Supplementary Material, further inquiries can be directed to the corresponding author.

## Ethics Statement

The animal study was reviewed and approved by the Institutional Animal Care and Use Committee at Shandong Provincial Hospital (Jinan, China).

## Author Contributions

LX, YG, and GW performed most of the experiments, analyzed, interpreted data, and wrote the manuscript. GS, WS, JL, JW, and XL helped to construct the mouse model and perform some experiments. MZ supervised the project and wrote the manuscript. All authors contributed to the article and approved the submitted version.

## Conflict of Interest

The authors declare that the research was conducted in the absence of any commercial or financial relationships that could be construed as a potential conflict of interest.

## Publisher’s Note

All claims expressed in this article are solely those of the authors and do not necessarily represent those of their affiliated organizations, or those of the publisher, the editors and the reviewers. Any product that may be evaluated in this article, or claim that may be made by its manufacturer, is not guaranteed or endorsed by the publisher.

## Abbreviations

AHN: adult hippocampal neurogenesis
BDNF: brain-derived neurotrophic factor
TrkB: tyrosine receptor kinase B
NT-3: neurotrophin-3
TrkC: tropomyosin receptor kinase C
PND: perioperative neurocognitive disorders
SGZ: subgranular zone
DG: dentate gyrus
NSCs: neural stem cells
NPCs: neural progenitor cells
DCX: doublecortin
BrdU: 5-bromodeoxyuridine
MWM: Morris Water Maze.
