# Supplementary material for: Inhibition of Adult Hippocampal Neurogenesis Plays a Role in Sevoflurane-Induced Cognitive Impairment in Aged Mice Through Brain-Derived Neurotrophic Factor/Tyrosine Receptor Kinase B and Neurotrophin-3/Tropomyosin Receptor Kinase C Pathways
Source: Front Aging Neurosci. 2022 Mar 4;14:782932. doi: 10.3389/fnagi.2022.782932 (PMC8931760; doi:10.3389/fnagi.2022.782932)
Supplement: Supplementary file 1 [file Data_Sheet_1.PDF]

Supplementary Material

Supplementary Table 1. Swimming speed of aged and adult mice in MWM test. (cm/s) (Mean ± S.E.M, n = 7/group)

	CON	1.5% Sevo	3.0% Sevo	p value
Aged mice	20.33±0.67	18.60±0.52	19.35±0.84	0.219
Adult mice	19.93±0.65	20.07±0.98	19.38±0.97	0.844

Supplementary Table 2. One-way ANOVA analysis of AHN in aged mice with BDNF or NT-3 microinjection. (n = 8/group)

	BDNF	NT-3
DCX+ cells	F = 5.08, p = 0.006	F = 6.36, p = 0.002
BrdU+ cells	F = 12.65, p = 0.001	F = 15.13, p = 0.001
BrdU+DCX+ cells	F = 13.14, p = 0.001	F = 16.92, p = 0.001

Supplementary Table 3. Statistical analysis of cognitive function in aged mice with BDNF or NT-3 microinjection. (n = 7/group)

	BDNF	NT-3
Escape latency*	F = 5.741, p = 0.005	F = 5.552, p = 0.005
Time spent in targeted zone	F = 3.544, p = 0.039	F = 5.591, p = 0.007

Target-crossing	F = 4.039, p = 0.025	F = 3.442, p = 0.042
Alternation triplet	F = 5.028, p = 0.018	F = 4.066, p = 0.035

* Two-way ANOVA with repeated measurements was used to analyze the difference of learning curves (based on escape latency) of different groups in the MWM. One-way ANOVA was used to analyze other aspects of behavior test.
