# Supplementary material for: Inhibition of Adult Hippocampal Neurogenesis Plays a Role in Sevoflurane-Induced Cognitive Impairment in Aged Mice Through Brain-Derived Neurotrophic Factor/Tyrosine Receptor Kinase B and Neurotrophin-3/Tropomyosin Receptor Kinase C Pathways
Source: Front Aging Neurosci. 2022 Mar 4;14:782932. doi: 10.3389/fnagi.2022.782932 (PMC8931760; doi:10.3389/fnagi.2022.782932)
Supplement: Supplementary file 2 [file Data_Sheet_2.PDF]

Supplementary Material

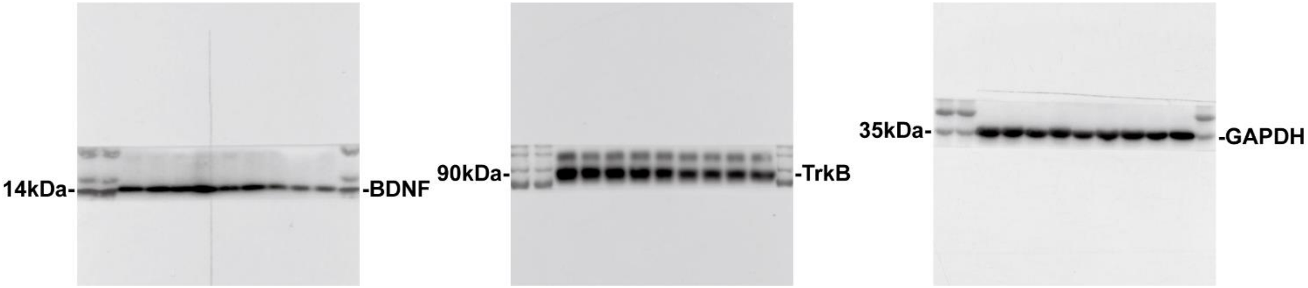

Supplementary Data 1. Full scan of the original blots in Figure 5b. From left to right: BDNF, TrkB, GAPDH.

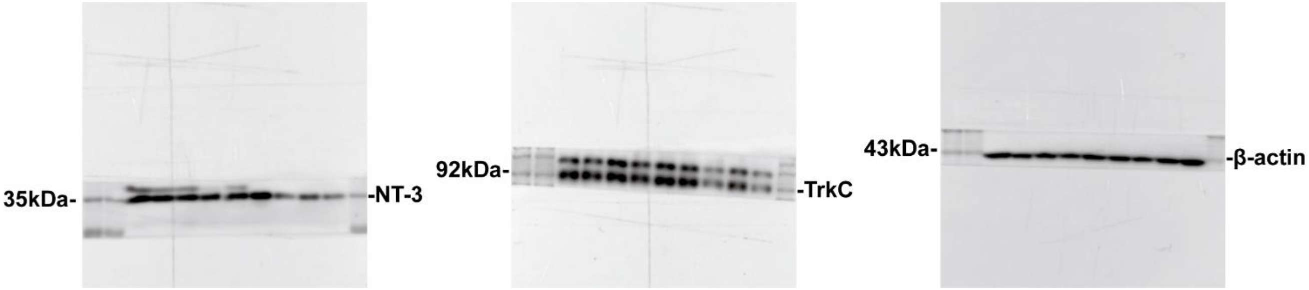

Supplementary Data 2. Full scan of the original blots in Figure 5e. From left to right: NT-3, TrkC, β-actin.

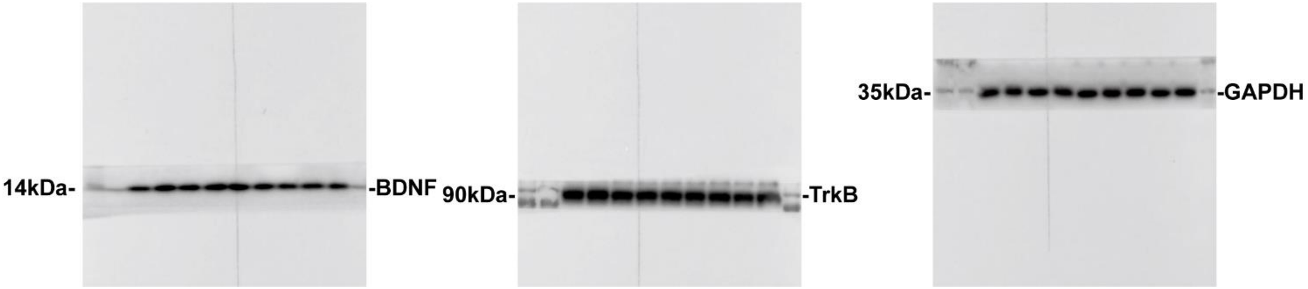

Supplementary Data 3. Full scan of the original blots in Figure 6a. From left to right: BDNF, TrkB, GAPDH.

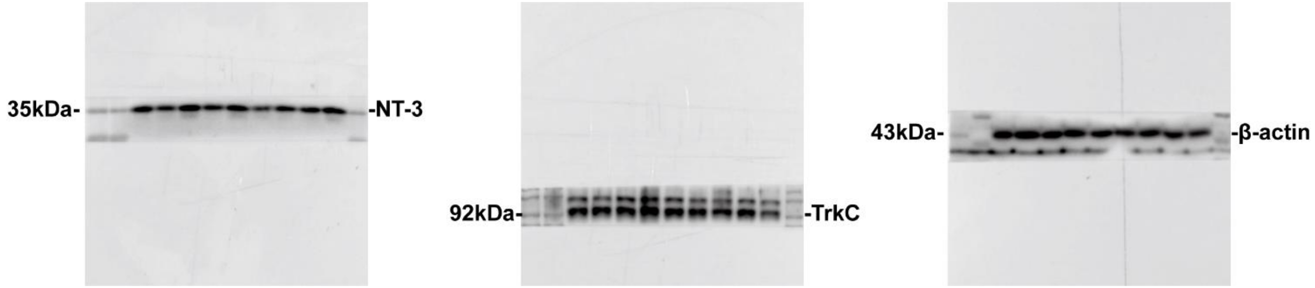

Supplementary Data 4. Full scan of the original blots in Figure 6d. From left to right: NT-3, TrkC, β-actin.
