# Supplementary material for: Inhibition of Adult Hippocampal Neurogenesis Plays a Role in Sevoflurane-Induced Cognitive Impairment in Aged Mice Through Brain-Derived Neurotrophic Factor/Tyrosine Receptor Kinase B and Neurotrophin-3/Tropomyosin Receptor Kinase C Pathways
Source: Front Aging Neurosci. 2022 Mar 4;14:782932. doi: 10.3389/fnagi.2022.782932 (PMC8931760; doi:10.3389/fnagi.2022.782932)
Supplement: Supplementary file 3 [file Data_Sheet_3.PDF]

Supplementary Material

Supplementary Figure 1. Representative immunofluorescence image of BrdU+DCX+ cell in hippocampal DG of aged mice exposed to 3% sevoflurane at 40X magnification.
