# Supplementary figures and images for: Inhibition of Adult Hippocampal Neurogenesis Plays a Role in Sevoflurane-Induced Cognitive Impairment in Aged Mice Through Brain-Derived Neurotrophic Factor/Tyrosine Receptor Kinase B and Neurotrophin-3/Tropomyosin Receptor Kinase C Pathways
Source: Front Aging Neurosci. 2022 Mar 4;14:782932. doi: 10.3389/fnagi.2022.782932 (PMC8931760; doi:10.3389/fnagi.2022.782932)

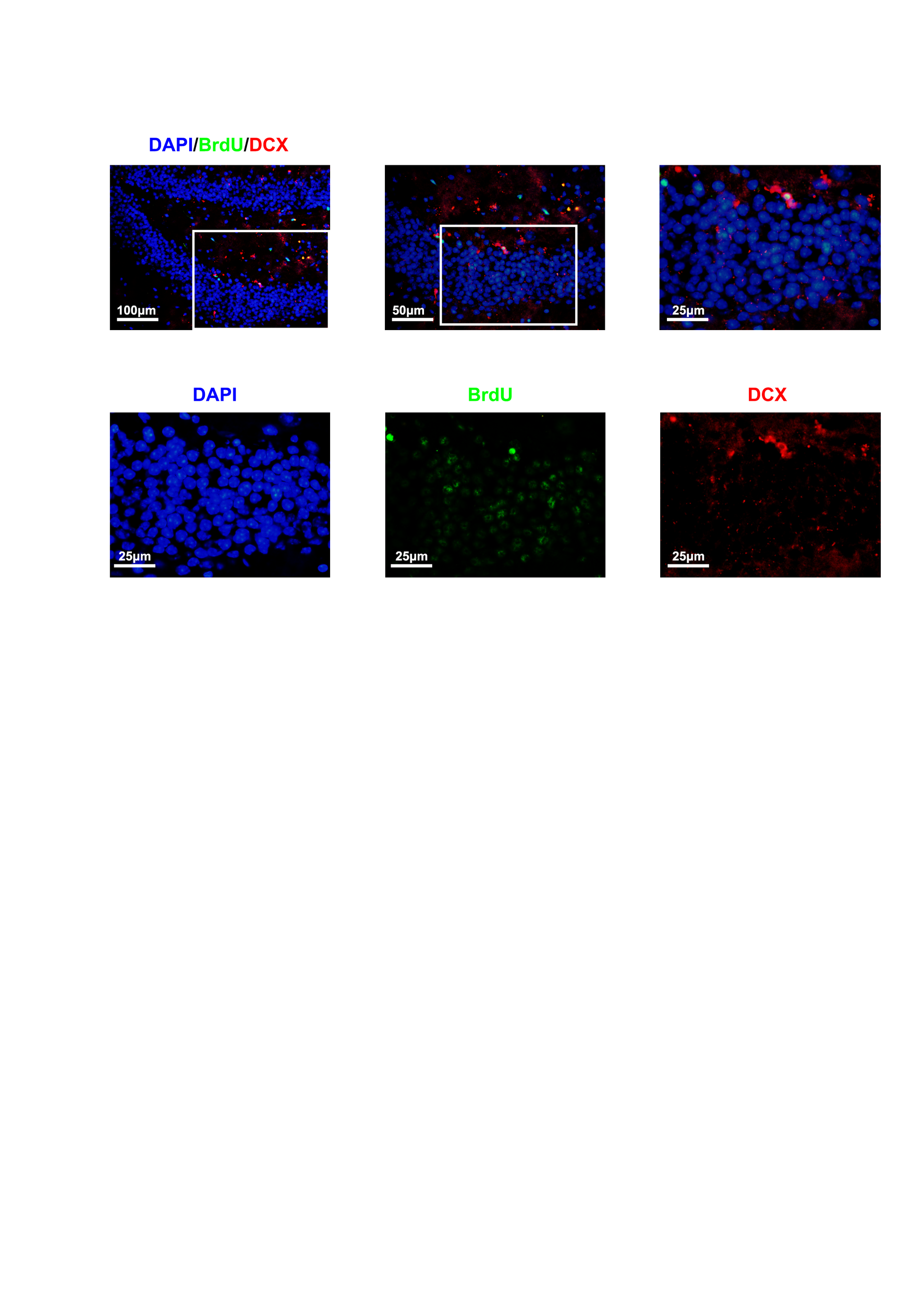

Supplement: Supplementary file 4 [file Image_1.TIF]
